# Enhancing Credibility of Chemical Safety Studies: No Consensus

**DOI:** 10.1289/ehp.1104130

**Published:** 2011-12-01

**Authors:** Tony Tweedale

**Affiliations:** R.I.S.K. (Rebutting Industry Science with Knowledge) Consultancy, Brussels, Belgium, E-mail: ttweed@base.be

I commend Conrad and Becker (2011) for frankly discussing the criteria by which to judge data quality in chemical risk assessment. Those criteria revolve tightly around financial conflict of interests. I agree with Conrad and Becker (2011) that industry seems likely to continue to perform the toxicity tests in risk assessment. That is due to what I call the “GLP [Good Laboratory Practices] shield.” Industry’s compliance with GLP has caused many tens of thousands of published independent chronic toxicity studies—any of which might have determined the allowable “safe” daily dose of a chemical—to be excluded from premarket approval risk assessments and substituted with financially conflicted, yet GLP-compliant, data from the chemical’s manufacturer.

It is incomprehensible to me that Conrad and Becker (2011) assert that a financial conflict of interests should not be a criterion in determining the financial independence of researchers (and therefore the reliability of results). A financial conflict of interests exists as soon as there is a link between a researcher and a monetary value. It does not signify unethical behavior, but it does warn of that possibility. Scientists should be reassured—not upset, as Conrad and Becker (2011) claim—if financial conflict of interests was the lead criteria to assess data quality. Conrad and Becker’s substitute criterion—disclosure of financial conflict of interests—becomes useless with their other recommendation to accept the data of financially conflicted scientists.

Conrad and Becker (2011) failed to mention the independent and consistent reviews that all but prove that sponsorship of science by the pharmaceutical industry produces results more financially favorable to them than those of financially independent science, and several reviews of toxicity studies of petrochemicals reach the same conclusion (Bekelman et al. 2003; Fagin et al. 1999; Swaen and Meijers 1988; vom Saal and Hughes 2005).

Repeatedly Conrad and Becker (2011) urge regulators to accept GLP as a key criterion determining data reliability (the ability to predict actual toxicities). The Organisation for Economic Co-operation and Development (OECD) creates toxicity study guidelines (OECD 2011) featuring GLP, which are adopted worldwide by regulators. These OECD regulatory test protocols are stuck in the age of the light microscope, test a narrow and unrealistic portion of the dose–response curve and relatively few end points, mostly fail to test toxicity during vulnerable development, and kill the animals being tested before most diseases develop (a human equivalent of ~ 60 years). Society should not accept that the OECD GLP protocols are better than those developed by independent, curious academics. Therefore, for any common petrochemical, readers should compare in depth the independent toxicity findings via PubMed and the OECD GLP alleged safe exposure level, which Conrad and Becker (2011) promote.

Conrad and Becker (2011) proposed that industry be allowed to continue to influence research, although they would discount studies for which a sponsor owns the results. Journals seem to prefer the simplicity and finality of forbidding outsider control of a researcher’s data.

Finally, Conrad and Becker (2011) (compared with Becker et al. 2009) gave lukewarm support to traditional journal peer review and publication, but they continue to question its value, claiming instead that peer review by government regulatory agencies is of better quality. However, such a criterion would simply reinforce these agencies’ current use of these financially conflicted data in determining risk assessment outcome. It would be better if risk assessment relied on traditional peer review, which is science’s most fundamental tool for ensuring reliable data. False-negative error is more consequential than false-positive error.
